# Starch Granules in *Arabidopsis thaliana* Mesophyll and Guard Cells Show Similar Morphology but Differences in Size and Number

**DOI:** 10.3390/ijms22115666

**Published:** 2021-05-26

**Authors:** Qingting Liu, Xiaoping Li, Joerg Fettke

**Affiliations:** Biopolymer Analytics, Institute of Biochemistry and Biology, University of Potsdam, Karl-Liebknecht-Str. 24-25 Building 20, 14476 Potsdam-Golm, Germany; qingting.liu@uni-potsdam.de (Q.L.); xiaopingli@uni-potsdam.de (X.L.)

**Keywords:** starch granules, starch metabolism, starch granule initiation, starch granule number per chloroplast, starch morphology, mesophyll cell, guard cell, LCSM, *Arabidopsis thaliana*

## Abstract

Transitory starch granules result from complex carbon turnover and display specific situations during starch synthesis and degradation. The fundamental mechanisms that specify starch granule characteristics, such as granule size, morphology, and the number per chloroplast, are largely unknown. However, transitory starch is found in the various cells of the leaves of *Arabidopsis thaliana*, but comparative analyses are lacking. Here, we adopted a fast method of laser confocal scanning microscopy to analyze the starch granules in a series of Arabidopsis mutants with altered starch metabolism. This allowed us to separately analyze the starch particles in the mesophyll and in guard cells. In all mutants, the guard cells were always found to contain more but smaller plastidial starch granules than mesophyll cells. The morphological properties of the starch granules, however, were indiscernible or identical in both types of leaf cells.

## 1. Introduction

Transitory starch is a major storer of carbon and energy, and it allows plants to overcome dark phases when photosynthesis is impossible. In recent years, huge progress has occurred toward understanding plastidial starch metabolism in the model plant *Arabidopsis thaliana* [1,2,3]. Generally, starch metabolism consists of two processes: synthesis and degradation. The initiation of starch granules has been a research focus in recent years, as it is considered the first step in starch synthesis, starting with a primer-like structure/initiation complex formation [4]. It determines or affects the features of the entire starch granule, such as size, number, and morphology. The precise mechanism for starch initiation has, however, not been clarified yet; many enzymes and proteins have been described that affect the quantity, distribution, and morphology of starch granules. For example, Arabidopsis mutants not expressing the starch synthase isoform 4 (SS4) revealed that most chloroplasts are devoid of any starch granules, but a small proportion of chloroplasts contain more roundly formed starch granules, whereas most chloroplasts of the mesophyll cells were found to be free of starch granules. For unknown reasons, this phenotype is especially pronounced in young leaves [5]. In contrast, in the wild type, four to seven starch granules per chloroplast with flattened and discoid shapes were detected [6]. Thus, SS4 is considered to play a central role in starch initiation [7]. Similarly, mutants lacking an interacting protein of SS4, protein targeting to starch 2 (PTST2), revealed that most of the leaf chloroplasts contain none or only one starch granule, which is larger but still typically flattened and discoid. The shapes of these starch particles are similar to those observed in the wild type [8]. In addition, the lack of both the cytosolic isoform of the disproporting enzyme (DPE2 [9,10,11]) and the plastidial isozyme of the phosphorylase (PHS1 [12,13]) led to a dramatically reduced granule number per chloroplast, mostly to one, as well as more spherical starch granules [6,13]. Surprisingly, the Arabidopsis triple mutant lacking in additional SS4 displayed even larger and nearly perfectly spherical starch granules [6]. However, the periods of starch synthesis and degradation were not completely separated. This conclusion was reached as multiple mutants lacking enzymes involved in starch degradation, such as *dpe2*, *sex1-8*, and *bam3*, displayed thicker and/or larger starch granules [13,14]. Moreover, mutants defective in the initiation process were also impaired in degradation, implying that the normal starch granule number is essential for proper starch remobilization during the night [5]. Therefore, such comprehensive alterations in size, quantity, and morphology reflect substantial changes in the starch metabolic system, which, in turn, increase the complexity of separating various starch parameters and decoding their underling mechanisms.

For comprehensive analyses of the starch granule parameters, which also entail the characterization of starches from various tissues and cells in their native environment, a simple and fast method is needed. Common is the use of electron microscopy (scanning electron microscopy (SEM) or transmission electron microscopy (TEM) [15]) and atomic force microscopy [16] for the detailed characterization of starch granules; however, these methods are complex with regards to sample preparation (e.g., sample extraction and metal coating and sectioning) and allow observation of the granules only outside of their native environment or under unnatural conditions. Additionally, for very limited tissues and cells, such as guard cells, these methods are unusable, because not enough starch particles can be prepared, and suitable sections cannot be analyzed.

However, laser confocal scanning microscopy (LCSM) is a widely used method to detect, e.g., subcellular structures, protein locations, and protein–protein interactions, relying on specific dyes as markers [17,18]. Here, we used LCSM combined with safranin O as a rapid and simple method to observe starch granules in their native environment.

At present, most data on the Arabidopsis transitory starch granule parameters are related to mesophyll cells, and we characterized their starch granules with this new method to prove both the reliability of the published parameters and the method. However, it is unknown whether the observed characteristics are also detectable in other cell types and tissues. In this regard, one interesting cell type is the guard cell, which has an independent and unique physiological function. Here, starch was found to play a key role in regulating the stomata. For stomata opening, the starch in guard cells is degraded, and the resulting inner osmosis pressure promotes the opening. In parallel, the ion pump in the plasma membrane is activated, which is triggered by blue light. The starch metabolism in guard cells is not fully understood but is obviously differently regulated compared with mesophyll cells. In the latter, starch degradation occurs during the night, but most starch in guard cells is remobilized within the first hour of dawn [19,20,21]. Furthermore, a rapid and flexible degradation of starch is essential for guard cells to react to the changing environment.

The starch granule number seems vital for regulating the starch breakdown rate, as, in wild-type mesophyll cells, the accumulated starch is almost completely degraded at dawn. However, it was reported that decreased starch granule numbers result in a lower starch turnover rate [6]. Mesophyll cells of the *ss4*, *dpe2/phs1*, *dpe2/phs1/ss4*, and *ptst2* mutants all showed higher starch contents at the end of night than the wild type [6,8]. However, it was unclear whether the guard cells produced similar alterations in starch granule parameters as those described for mesophyll cells. Moreover, it would be interesting to identify how the guard cells cope with the contradiction between the demand for the flexible degradation of starch and a possible decreased starch granule number.

Using our method, we revealed a decrease in the starch granule number in mutants such as *ss4*, *dpe2/ss4*, *phs1/ss4*, *dpe2/phs1/ss4*, and *dpe2phs1*, and the morphology was similar for both mesophyll and guard cells; however, guard cells possessed slightly more starch granules than the corresponding mesophyll cells. This may indicate that, in guard cells, the starch degradation, depending on the granule number, is more conserved compared with mesophyll cells when adapting to its unique physiological function.

## 2. Results

### 2.1. Starch Granule Analyses in Arabidopsis thaliana Mesophyll Cells via LCSM

**Figure 1:**
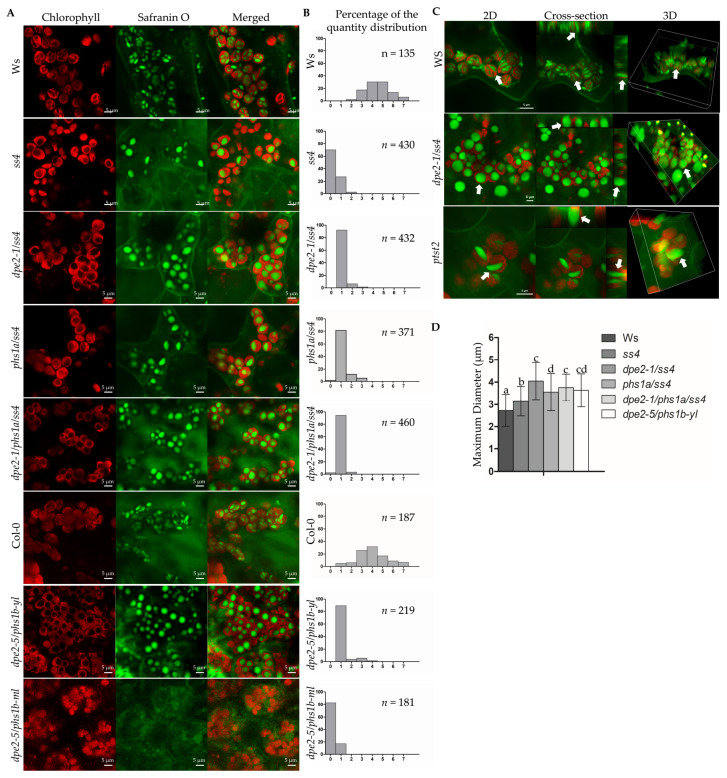
Starch granules in mesophyll cells. (**A**) Graphs were taken from 3-week-old rosette leaves of wild types and mutants. All samples were taken in the middle of the light phase. *Dpe2-5/phs1b* accession is Col-0; yl, young leaves and ml, mature leaves. Scale bar is equivalent to 5 μm. (**B**) The quantity distribution of the starch granules per chloroplast in the mesophyll cells. (**C**) The 2D, cross-section, and 3D views of the starch granules isolated from Ws, *dpe2-1/ss4*, and *ptst2*. Scale bar as indicated. (**D**) Diameters of the starch granules in the mesophyll cells, measured by ZEN 2 lite software, *n* > 80. The mean and SD are shown. Letters indicate statistically significant differences (*p* ≤ 0.05) according to the one-way ANOVA with Tukey’s post-hoc test.

Our method is a very fast procedure that requires only a few minutes for preparation and, therefore, facilitates the analyses of many samples. As reported, the starch granules in ss4 were more spherical and oval; in *dpe2/ss4* and *dpe2/phs1/ss4*, the granules were larger and nearly spherical-shaped; in contrast, the wild-type starch granules were flattened, discoid, and small (Figure 1A). The double-mutant *dpe2/phs1* revealed the reported alteration in the starch granule number per chloroplast in young and mature leaves, with young leaves having mostly one starch granule per chloroplast, whereas mature leaves showed disintegration of the chloroplasts and mostly lacked starch granules (Figure 1A; [22]). We also confirmed the discovery that most chloroplasts in *ss4* have no or only one single starch granule. In contrast, a lack of DPE2, PHS1, or DPE2/PHS1 in the *ss4* background resulted in an increase in the number of granules to mostly one or two starch granules in the mesophyll cells (Figure 1B).

The LCSM analyses facilitated the fast and clear inspection of the three-dimensional shape and thereby avoided the misinterpretation of granule morphology that may occur when using only 2D pictures. Figure 1C shows 3D and cross-section pictures of the wild type, *ptst2*, and *dpe2/ss4*. Typically, the starch granules of the wild type and *ptst2* were flattened and discoid. For *ptst2*, larger starch granules (5.20 ± 0.74 µm, *n* = 20) compared to the wild type were observed, as reported previously [8]. In *dpe2/ss4*, the starch granules were exactly spherical (as revealed from the vertical view and cross-section view, as well as the 3D view), in contrast with those of the wild type and *ptst2*. The sizes of the various starch granules were also determined using ZEN 2 lite to ensure that the new method was able to depict the known alterations in the sizes of the granules (Figure 1D). The starch granules of *ss4* (3.1 ± 0.6 µm) were significantly larger than those in the wild type (2.7 ± 0.7µm). The starch granules of the double and triple mutants—lacking PHS1, DPE2, or both proteins, in addition to SS4—were approximately 3.6–4.0-µm significantly larger than those of the single-mutant *ss4*. The granule size in the young leaves of *dpe2/phs1* (3.6 ± 0.7 µm) was more similar to the sizes of the double and triple mutants than that of the single-mutant *ss4*.

### 2.2. The Morphology of Starch Granules in Guard and Mesophyll Cells Was Similar, but the Granule Size Was Altered

**Figure 2:**
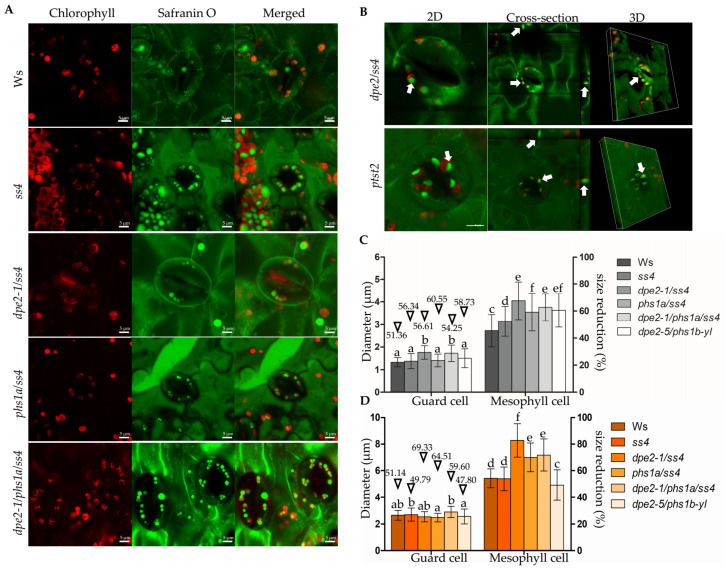
Starch granules in the guard cells. (**A**) Graphs were taken from 3-week-old rosette leaves in the middle of the light phase. Scale bar is equivalent to 5 μm. (**B**) The 2D, cross-section, and 3D views of the starch granules of *dpe2/ss4* and *ptst2*. White arrows mark the target starch granule. (**C**) Diameters of the starch granules in the guard and mesophyll cells, *n* > 100. (**D**) Diameters of the chloroplasts in the guard and mesophyll cells, *n* = 100. (**C**,**D**) The mean and SD calculated from 50 values. Letters indicate the statistically significant differences (*p* ≤ 0.05) according to the one-way ANOVA with Tukey’s post-hoc test. Inverted triangles and corresponding values indicate the percentage of the size reduction in the starch granules (**C**) and chloroplasts (**D**) in the guard cells compared with the mesophyll cells.

Starch granules of wild-type guard cells were easily detected and revealed the same shape as those observed for the mesophyll cells. Similar to *ss4*, *phs1a/ss4*, *dpe2/ss4*, and *dpe2/phs1/ss4*, typical morphology was observed. Thus, they displayed more rounded or spherical-shaped granules (Figure 2A). We analyzed two other mutants that differed significantly in starch morphology in the mesophyll cells: *dpe2/ss4* and *ptst2*. *dpe2/ss4* showed typical round and *ptst2* flat and larger starch granules (Figure 1C). Even the starch granules in the guard cells were much smaller; the overall morphology was still detectable and similar to that of the mesophyll cells (Figure 2B). Thus, the guard cell starches of *ptst2* and *dpe2/ss4* were also long and flat or round and more spherical, respectively.

For the wild type and all mutants, the starch granule size in the guard cells was approximately half that in the corresponding mesophyll cells (51% to 61%; Figure 2C). Interestingly, the pattern of the granule size difference was also similar; thus, the same alterations in size that were observed for the mesophyll cells between the various mutants were detected in the guard cells. Therefore, the bigger starch granules observed in the mesophyll cells were also detected as bigger in the guard cells, even though the proportion was not the same for all analyzed mutants.

**Figure 3:**
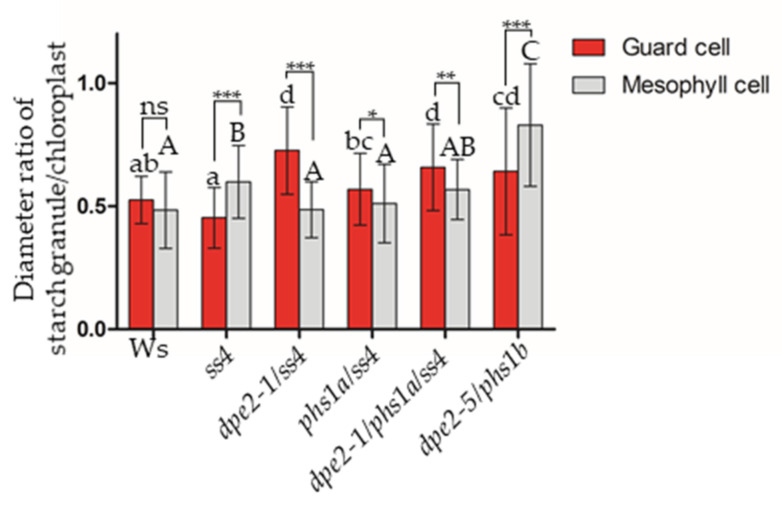
Relative size ratio of the individual starch granules and chloroplasts. The ratios of the starch granule sizes and chloroplast sizes in the guard and mesophyll cells are provided. The mean and SD calculated from 50 values are shown. Upper- and lowercase letters indicate the statistically significant differences (*p* ≤ 0.05) among the mesophyll and guard cells, respectively, according to the one-way ANOVA with Tukey’s post-hoc test. Asterisks show the significant differences between the mesophyll and guard cells within the same lines: ns, no significance; * *p* < 0.05, ** *p* < 0.01 and *** *p* < 0.001, based on *t*-tests.

In the mesophyll cells, the pattern of the starch granule sizes largely followed that of the chloroplasts, especially in mutants that contained one starch granule per chloroplast. Larger starch granules were coupled with larger chloroplasts (Figure 2D). However, the chloroplast sizes were altered less in the guard cells compared with the mesophyll cells. *phs1/ss4*, *dpe2/ss4*, and *dpe2/phs1/ss4* revealed the biggest mesophyll chloroplasts, around 7.0–8.0 µm. The corresponding starch granules were, at 3.5–4.0 µm, also the biggest in the mesophyll cells. In the guard cells, these three mutants showed comparable chloroplast sizes to all the other plant lines, but the starch granule sizes, especially of *dpe2/ss4* (1.75 ± 0.30 µm) and *dpe2*, *phs1*, and *ss4* (1.72 ± 0.36 µm), were significantly larger. Therefore, we excluded the idea that the granule size was only linked to the chloroplast size, particularly for the situation in the guard cells. Furthermore, when comparing the ratios of individual starch with chloroplast sizes, it was obvious that the lack of SS4, together with DPE2, PHS1, or both, resulted in bigger relative starch granule sizes in the guard cells, whereas the lack of SS4 alone, as well as missing both DPE2 and PHS1, revealed higher relative starch sizes in the mesophyll cells (Figure 3). Thus, the various mutations differently affected the starch-to-chloroplast size ratio in the two cell types. Interestingly, in *dpe2/phs1*, both the relative starch granule sizes of the guard and mesophyll cells were higher compared with the wild type.

### 2.3. Guard Cells Contained More Starch Granules Than Corresponding Mesophyll Cells of ss4, dpe2/ss4, phs1/ss4, and dpe2/phs1/ss4 Mutants

**Figure 4:**
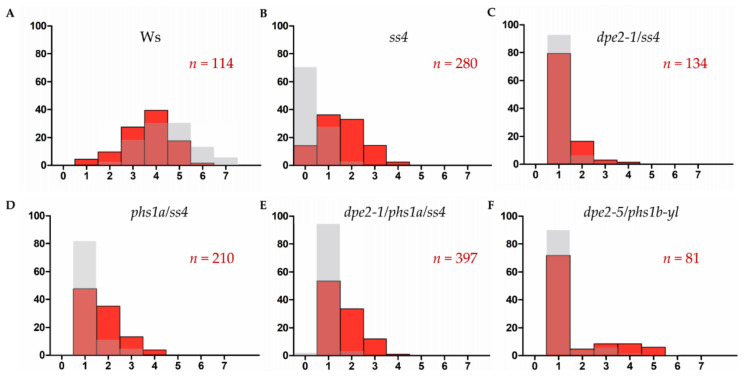
Distribution of the starch granule number per chloroplast. The quantity of the starch granules per chloroplast in the guard cells (red) was determined and compared with that of the mesophyll cells (grey). yl, young leaf. **A**–**F** indicate the data from wild type and corresponding mutants, respectively.

A substantial amount of starch granules in the guard cells was analyzed. The starch granule number of the wild-type plants was determined with a typical distribution of one to seven granules per chloroplast, with four being most common (Figure 4A). However, compared with the mesophyll cells, the distribution pattern of starch granules in the guard cells was slightly shifted to a lower granule number. In contrast, all mutants showed more starch granules in the guard cells than the mesophyll cells. However, no wild-type level was observed. Except for *dpe2/phs1*, for all the other mutants, the maximum number of starch granules per chloroplast was restricted to four. Remarkably, all the analyzed chloroplasts, independent of the mesophyll or guard cells, always contained at least one starch granule (Figure 4C–F), in contrast with the situation in *ss4*, where the lack of granules was also detected. Furthermore, the granule number distribution of *ss4* shifted and was similar to the wild-type pattern compared with that of the other analyzed mutants.

**Figure 5:**
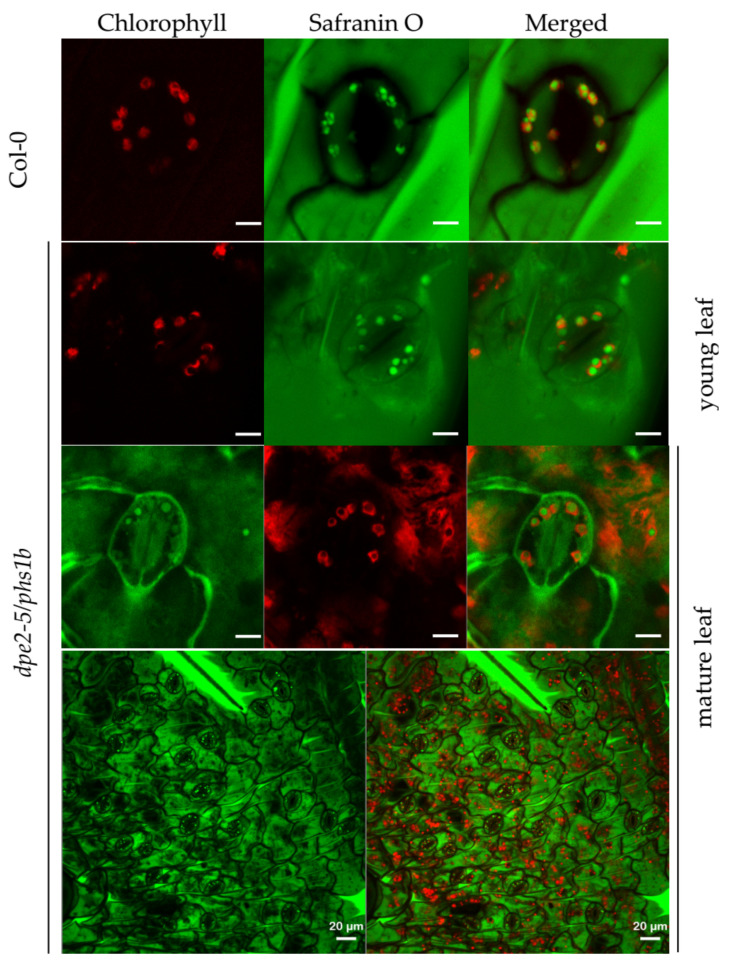
Starch granules in the guard cells of Col-0 and young and mature *dpe2-5/phs1b* leaves. All pictures were captured from 5-week-old plants in the middle of the daytime. Scale bars are equivalent to 5 and 20 μm.

The higher starch granule number in the guard cell chloroplasts did not result in a reduction in the relative starch sizes (Figure 3); in contrast, a higher relative size was observed for *dpe2ss4, phs1ss4,* and *dpe2phs1ss4*. For *ss4* and *dpe2/phs1* (only one starch granule per chloroplast; Figure 5), the impact on the starch granule size was the opposite (Figure 3). However, the size ratios in the wild type were the same.

### 2.4. In the Double-Mutant dpe2/phs1, the Alterations in Starch Granule Number per Chloroplast in Young and Mature Mesophyll Leaf Cells Were Not Observed in the Guard Cells

Previous studies showed that the biochemical processes in mesophyll cells change with leaf age. Thus, the double-mutant *dpe2/phs1* displayed an uneven distribution of starch, and the detectable starch was restricted to young leaves [3,10]. Most of the mesophyll cell chloroplasts in young leaves had only a single and nearly perfect spherically shaped starch granule, whereas, in mature leaves, there were no detectable starch granules, and the chloroplasts were mostly disintegrated [3].

We observed starch granules in the young and mature leaves of *dpe2/phs1* separately to test whether this was also the case for the guard cells. Due to the different backgrounds of the mutants, we analyzed the starch granules in Col-0, which showed the same typical shape and a similar quantity compared to Ws for both the mesophyll and guard cells (Figure 1 and Figure 5). We confirmed that most mesophyll cell chloroplasts from young leaves contained only one starch granule. Notably, for the guard cells, similar to the other mutants, a slightly higher granule number per chloroplast was observed. All the chloroplasts revealed spherical starch granules. As expected, in the mesophyll cells of the mature leaves, there were only a few verifiable starch granules, and most chloroplasts were disintegrated. Surprisingly, the guard cells of the mature leaves still showed only one spherical starch granule. This was a higher starch granule number compared with the mesophyll cell situation. However, the existence of at least one starch granule in the guard cells is not unexpected, as the living mature leaves still need to regulate their gas exchange and water evaporation.

## 3. Discussion

### 3.1. A Rapid and Simple Method for Starch Granule Analyses in Their Native Environment

In this study, we adopted LCSM and safranin O staining to observe starch granules in mesophyll and guard cells and determined the quantity, size, and shape of the starch granules of several known starch-related mutants (Figure 1A and Figure 2A). Overall, it is a fast, simple, and reliable method for characterizing the center of starch metabolism—the starch granules. Relying on 3D and cross-section views, starch granules with totally different morphologies in *dpe2/ss4*, *ptst2,* and the wild type were effectively characterized (Figure 1C and Figure 2B). A limitation of TEM or any other technique that relies on sectioning samples, regarding the spatial distribution of starch granules in chloroplasts, is the inaccurate estimation of the number of starch granules per chloroplast. Thus, analyzing a single section of a chloroplast leads to an underestimation of the exact number. However, this new established method allows the consecutive detection of the chloroplast and, therefore, determination of the exact starch granule number.

Compared with the complex and time-consuming sample preparation required for SEM and TEM, this method only needs several minutes for staining and allows the analysis of starch granules in chloroplasts from different tissues or cell types. Thus, a much higher number of samples can be examined, and the time resolution of the analyses can easily be increased. Furthermore, even small size differences such as, e.g., alterations in the starch granule sizes of guard cells, are clearly detectable.

### 3.2. The Number of Starch Granules per Chloroplast in Guard and Mesophyll Cells Was Affected by the Lack of the Same Proteins but Differently Strict

All the analyzed mutants displayed similar changes in the starch granule initiation and granule morphology for both the mesophyll and guard cells. This illustrates the similar roles of SS4, PHS1, DPE2, and PTST2 in both cell types. Thus, the starch granule morphology was always the same in both cell types. However, the guard cells were slightly less affected by alterations in the starch granule numbers, as the guard cells contained more starch granules compared with the corresponding mesophyll cells. However, except for *ss4* and *dpe2/phs1*, the relative diameter ratio of starch granule(s)/chloroplast in the guard cells was higher than that observed in the mesophyll cells. Thus, for *dpe2/ss4*, *phs1/ss4*, and *dpe2/phs1/ss4*, each chloroplast of the guard cells revealed a higher starch content compared with the mesophyll cell situation. Regarding the granule number distribution, in the wild type, there was a small shift in the pattern toward smaller amounts in the guard cells compared with the mesophyll cells. The granule number distribution was totally different in the case of *ss4* and the double and triple mutants. The *ss4* distribution pattern showed similarities to that of the wild type but shifted to higher granule numbers. In contrast, for the double and triple mutants, an asymptotic granule number distribution was observed in both the mesophyll and guard cells (Figure 4). Interestingly, in the guard cells of all the mutants (except *dpe2/phs1*), the maximum number of starch granules per chloroplast was four, reaching up to six in the wild type (Figure 4). One possible explanation is that, in all mutants, the capacity to initiate starch granules was reduced; this also indicates that more than one pathway exists for starch granule initiation. Furthermore, as the starch granule number is essential for proper starch remobilization in the guard cells and, thereby, for stomatal opening, the higher granule number in the guard cells compared with the mesophyll cells may reflect a metabolic compensation.

The alteration to an increased number per chloroplast in the guard cells compared with the mesophyll cells and the simultaneous conversation of the number distribution point to the same mechanistic regulation but to a different amount of strictness in the control. Possibly, this will allow these regulatory parameters to be detected in the future.

### 3.3. dpe2/phs1 Is Unique in Starch Granule Characteristics

*dpe2/phs1* revealed peculiar starch granules. In young leaves, the typical more spherical and larger granules were detected. In mature leaves, most chloroplasts were disintegrated and free of starch granules (Figure 1A, [13,22]). However, in contrast with the disintegration of chloroplasts and the loss of starch granules in mesophyll cells, the guard cells still had an intact chloroplast with typically one starch granule (Figure 4F and Figure 5). Additionally, the granule number distribution was unique. A tendency of one granule per chloroplast was detected in the guard cells, but they also had a distribution similar to the wild type, though with a much lower intensity. Perhaps this reflected the influences of two overlapping pathways for starch initiation, as discussed by Malinova et al. (2018) [4]. As reported, the starch in guard cells undergoes rapid degradation in response to light at the start of the day [19,20,21]. Our results support the idea that starch in guard cells serves as a guarantee for adequate glucose provisioning, which is used for stomatal opening, thereby maintaining the normal function of guard cells. In this regard, even in *ss4*, guard cell chloroplasts without starch granules were detected but never in all chloroplasts from one single guard cell. Thus, no guard cell was entirely free of starch granules.

However, *dpe2/phs1* was the only analyzed mutant that revealed an alteration in the relative starch sizes in both the mesophyll and guard cells and, therefore, again, showed a specific regulation of starch granule formation that was completely different from all other mutants with altered starch granule numbers. Interestingly, the lack of SS4 in the double mutant also eliminated these specific characteristics. Thus, the strict regulation of the granule number to mostly one granule in the guard cells, as well as the shape of the starch granules in both the mesophyll and guard cells, were not detected in *dpe2/phs1/ss4* (Figure 4 and Figure 5).

### 3.4. The Morphology of Starch Granules Was Not Affected by Granule Number or Size and Was the Same in Mesophyll and Guard Cell Chloroplasts

For all starch granules analyzed, the typically described morphology was observed in the case of both the mesophyll and guard cells. However, in the guard cells, the starch granules were smaller. The granule size differences in the various plant lines compared with the situation in the mesophyll cells were determined to be 51–60% (Figure 2C). However, the granule size differences observed among the various mutants were similar for both cell types.

However, there was no clear link between the starch granule size and the chloroplast size, especially for *dpe2/ss4* and the young leaves of *dpe2/phs1* (Figure 3). It should be considered that the correct volume of both was not determined; instead, the biggest dimension of the starch granules and chloroplasts was measured. Thus, a connection between the chloroplast volume and starch granule size is unlikely but cannot be excluded. However, no connection between the chloroplast size and the number of starch granules was detected as reported by Crumpton-Taylor et al. (Figure 2D) [23]. The chloroplast size of, e.g., the wild type, *ss4*, and *dpe2/phs1* (Ws: 5.4 ± 0.6 µm, *ss4*: 5.4 ± 0.8 and *dpe2/phs1*: 4.9 ± 0.6 µm), was unaltered, but the starch granule number massively differed from a lack of granules to the detection of up to seven granules (Figure 1B and Figure 4).

For all the analyzed starch granules, the overall morphology seemed to be the same in the guard and mesophyll cells. This was also tested for the long and flat *ptst2* starch granules (Figure 1C and Figure 3).

The data clearly revealed that the starch parameters, such as the granule number per chloroplast, the size, and the morphology of the starch granules, were independently influenced in the mutants, and no direct link was identified. Additionally, no connection between the granule number or size and the amount of stored carbohydrate was observed; thus, a simple carbon–reserve connection can be excluded.

We described a system where the starch morphology is uncoupled from the size and number, which is the first step to identifying the underlying regulatory mechanisms. Interesting, in this regard, is the detected starch granules in the guard cells of mature *dpe2/phs1* leaves. As mesophyll cells mostly show no starch, the control of starch granule initiation and formation in both cell types has to be differently regulated; thus, this double mutant is a promising model to illuminate these important processes.

## 4. Materials and Methods

### 4.1. Plant Materials and Growth Conditions

*Arabidopsis thaliana* seeds were sterilized by 6% (*v*/*v*) hypochlorous acid with 0.02% (*v*/*v*) Tween-20 and sown on 0.8% (*w*/*v*) agar plates with Murashige–Skoog (MS) medium, 1% (*w*/*v*) sucrose, and 0.05% (*w*/*v*) MES, pH 5.7. The plates were transferred to darkness at 4 °C for three days. Seedlings were transplanted to soil after one week of growing in an illumination incubator with a light/dark regime (light: 23 °C, 60% humidity, 12 h; dark: 18 °C, 60% humidity, 12 h). Plants were grown in chambers under the same conditions until sampling.

The *ss4*, *dpe2/ss4*, *phs1a/ss4*, and *dpe2/phs1/ss4* mutants in the Wassilewskija-0 background were described previously [5,6,13]. The double-mutant *dpe2-5/phs1b* in the Columbia-0 background was reported by Malinova et al. [13].

The T-DNA insertion mutant of PTST2 (*SALK_206998*; *Col-0*) was obtained from the Salk Institute Genomic Analysis Laboratory (La Jolla, CA, USA). Homozygosity was confirmed by PCR (forward primer (*Lp*): *5-GAGACAACTGCTGGGAACTTGGAGA-3* and reverse primer (*Rp*): *5-GCAATAAGCCAATTGTTTACATGCCTC-3*; T-DNA-specific primer *LBa1*: *5-TGGTTCACGTAGTGGGCCATCG-3*), see Supplemental Figure S1.

### 4.2. Safranin O Staining

Safranin O (Sigma, S8884,Munich, Germany) was dissolved in water (5 mg/mL), as previously reported [24]; the whole plants or excised leaves were harvested at the indicated time points and submerged into a safranin O solution for 0.5–1 h, allowing the dye to enter the tissue from the stomata and incision. Next, the material was washed with water to remove the surplus dye. The stained starch granules were visualized in the background of chloroplast auto-fluorescence with a strong contrast by selecting the proper pseudo-color.

### 4.3. Laser Confocal Scanning Microscopy

Images were obtained on a Zeiss LSM 880 Airyscan confocal microscope (Carl Zeiss, Jena, Germany), with a 63× oil-immersion lens with a 1.4 numerical aperture. Safranin O signals and auto-fluorescence of chlorophyll were monitored using an argon laser at a 488-nm wavelength for excitation, and the emitted light was captured on the spectral detector array in a wavelength window of 410–696 nm. The absorption peak of safranin O was from 510 to 690 nm and that of chlorophyll from 640 to 696 nm. Safranin O and chlorophyll autofluorescence were separated using online spectral fingerprinting. The diameters and quantities of the starch granules were measured using a ZEN lite 2.

## Data Availability

Data is contained within the article or Supplementary Materials.

## Supplementary Materials

The following are available online at https://www.mdpi.com/article/10.3390/ijms22115666/s1.
