# Supplementary material for: Starch Granules in Arabidopsis thaliana Mesophyll and Guard Cells Show Similar Morphology but Differences in Size and Number
Source: Int J Mol Sci. 2021 May 26;22(11):5666. doi: 10.3390/ijms22115666 (PMC8199161; doi:10.3390/ijms22115666)
Supplement: Supplementary file 1 [file ijms-22-05666-s001.zip › ijms-1192949-supplementary.pdf]

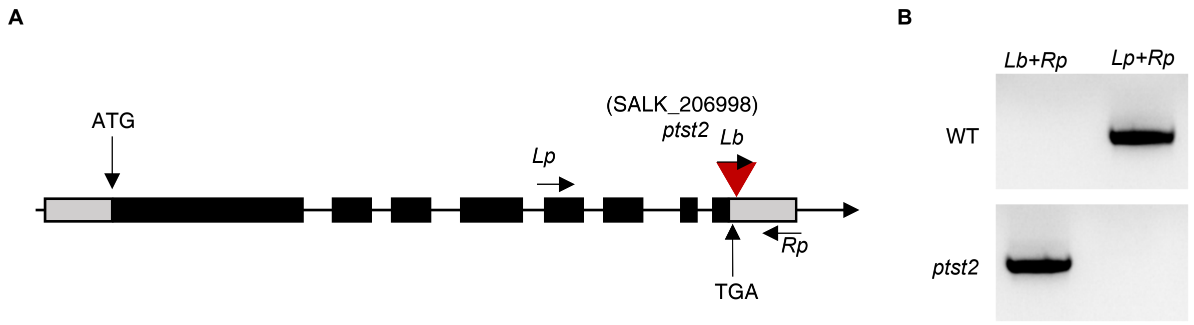

Supplemental figure 1. Confirmation of PTST2 T-DNA insertion mutant (**A**) Insertion site as referred to sequence information of TAIR database. (**B**) Confirmation of *ptst2.* Primers as indicated in (**A**).
